# Control of IGF-I levels with titrated dosing of lanreotide Autogel over 48 weeks in patients with acromegaly

**DOI:** 10.1111/j.1365-2265.2008.03208.x

**Published:** 2008-08

**Authors:** Philippe Chanson, Françoise Borson-Chazot, Jean-Marc Kuhn, Joëlle Blumberg, Pascal Maisonobe, Brigitte Delemer, the Lanreotide Acromegaly Study Group

**Affiliations:** * Université Paris-Sud 11 and Assistance Publique-Hôpitaux de Paris, Endocrinology and Reproductive Diseases, Bicêtre Hospital, and INSERM U693Le Kremlin-Bicêtre, France; † Nuclear Medicine, Neurological HospitalLyon, France; ‡ Endocrinology and INSERM CIC 0204, Rouen University HospitalFrance; § Medical Department, Ipsen LtdParis, France; ¶ Endocrinology, Maison Blanche HospitalReims, France

## Abstract

**Background:**

An essential criterion for control of acromegaly is normalization of IGF-I levels. Somatostatin analogues act to suppress IGF-I and GH levels.

**Objective:**

To assess the efficacy and safety of 48 weeks titrated dosing of lanreotide Autogel.

**Design:**

Open-label, multicentre, phase III, 48-week trial.

**Methods:**

Patients with active acromegaly (IGF-I levels > 1·3 times upper limit of age-adjusted normal range) were recruited. Twelve injections of lanreotide Autogel were given at 28-day intervals: during the 16-week fixed-dose phase, patients received 90 mg; in the 32-week dose-titration phase, patients received 60, 90 or 120 mg according to GH and IGF-I levels. Intention-to-treat analysis was performed to determine the proportion of patients with normalized age-adjusted IGF-I levels at study end. Secondary evaluations included GH levels, clinical acromegaly signs and safety.

**Results:**

Fifty-seven of 63 patients completed the study. Lanreotide Autogel resulted in normalized age-adjusted IGF-I levels in 27 patients (43%, 95% CI 31–55). Mean GH levels decreased from 6·2 to 1·5 µg/l at study end, with 53 of 62 patients (85%) having GH levels ≤ 2·5 µg/l (95% CI 76·7–94·3) and 28 of 62 patients (45%) with levels < 1 µg/l (95% CI 32·8–57·6). Twenty-four (38%) had both normal IGF-I levels and GH levels ≤ 2·5 µg/l. Acromegaly symptoms reduced significantly in most patients throughout the study. The most common adverse events were gastrointestinal, as expected for somatostatin analogues.

**Conclusions:**

Using IGF-I as primary end-point, 48 weeks lanreotide Autogel treatment, titrated for optimal hormonal control, controlled IGF-I and GH levels effectively, reduced acromegaly symptoms and was well tolerated.

## Introduction

Acromegaly is a chronic disease characterized by increased circulating levels of GH and IGF-I. Normalization of IGF-I levels is the main objective for the control of acromegaly,^1^ and is associated with improved cardiac function^2^ and reduction in excess mortality.^3^,^4^

Suppression of GH and IGF-I can be achieved by treatment with somatostatin analogues, which provide safe and effective management of acromegaly.^5^–^7^ The somatostatin analogue lanreotide is available as a long-acting aqueous gel formulation, lanreotide Autogel (Beaufour Ipsen Pharma, Paris, France), which is administered subcutaneously every 28 days from a ready-to-use prefilled syringe. Previous studies have demonstrated the good efficacy of lanreotide Autogel in the management of patients with acromegaly.^8^–^12^ Differing sensitivities to somatostatin analogues among patients with acromegaly mean that the doses of lanreotide required to normalize serum IGF-I levels will vary.^9^ Thus, individualizing dosing relies on appropriate dose titration for optimal control of serum IGF-I levels.^9^ The aim of this open-label study was to evaluate the efficacy of repeated injections of lanreotide Autogel for 48 weeks in a large cohort of patients with acromegaly. To allow comparison with other studies evaluating the effects of somatostatin analogues, as well as those assessing GH antagonists,^13^,^14^ the percentage of patients with a normal age-adjusted serum IGF-I level was chosen as the primary outcome. Secondary efficacy evaluations included the assessment of GH levels and clinical acromegaly signs. The safety of treatment was also documented.

## Patients and methods

### Patients

Patients (aged ≥ 18 years) were recruited if they had active acromegaly, defined as IGF-I levels at least 1·3 times the upper limit of the age-adjusted normal range. Patients who had previously received either a somatostatin analogue (other than lanreotide Autogel) or a dopaminergic agonist could be included if this elevated level of IGF-I was reached during the wash-out period, which lasted up to 12 weeks, depending on treatment previously administered. All somatostatin analogue or dopaminergic agonist treatment had to be discontinued at or before the first screening visit. A second screening visit was then scheduled as follows: 1 week after last administration of short-acting formulations of octreotide or dopaminergic agonist; 4 weeks after last administration of lanreotide 30 mg or long-acting dopaminergic agonist; and 8 weeks after last injection for long-acting octreotide formulation. If necessary, a third screening visit was scheduled 1 week after the previous visit for short-acting formulations, and 4 weeks after the previous visit for all others. If enrolment criteria were fulfilled at either of these visits, the patient was included. Patients were excluded from the study if they had previously received treatment with lanreotide Autogel. Patients were not permitted to have undergone pituitary surgery within the previous 3 months or have received radiotherapy for acromegaly within the previous 36 months.

All patients gave written, informed consent, and the study was approved by the institutional ethics committee of each study centre. The study was conducted in accordance with the Declaration of Helsinki (South Africa, 1996).

### Study design

This was an open-label, 48-week, phase III, multicentre study carried out by 17 investigators (16 in France and 1 in Switzerland). The study is registered on ClinicalTrials.gov (NCT00210457).

### Interventions

**Fig. 1:**
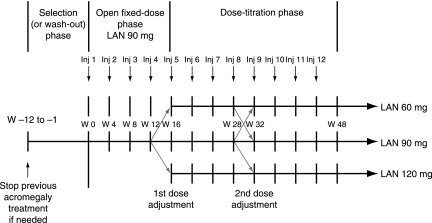
Study design schema.

Patients received a total of 12 injections of lanreotide Autogel at 28-day intervals over two study phases: four injections in the fixed-dose phase and eight injections in the dose-titration phase. During the fixed-dose phase, all patients received 90 mg of lanreotide Autogel, and in the dose-titration phase patients could receive 60, 90 or 120 mg. Dose adjustments occurred at weeks 16 and 32 according to patients GH and IGF-I levels as assessed at the preceding visit (Fig. 1). The total duration of the treatment period was 48 weeks.

The dose was increased if GH levels were > 2·5 µg/l or IGF-I levels were above the upper limit of the age-adjusted normal range, and decreased if GH levels were ≤ 1 µg/l and IGF-I levels were normal or below normal. At each dose titration, the dose could only be increased or decreased one level and, once increased to 120 mg, the dose could not be decreased later.

### Assessments and outcome measures

Blood samples were taken at 12, 28 and 48 weeks for evaluation of GH and IGF-I levels, and haematology and biochemistry measurements. GH and IGF-I levels were measured centrally. Tumour volume was not measured.

A set of seven blood samples was gathered at 30-min intervals for GH assessment at weeks 12 and 28, the last sample was taken just prior to lanreotide Autogel injection. GH levels were measured in a central laboratory using a GH immunoradiometric assay (Immunotech 1397, Marseille, France) with a detection limit of 0·05 µg/l. Maximal intra- and interassay coefficients of variation were 1·5% and 14%, respectively.

For assessment of IGF-I levels, a single blood sample was collected at the same time as the first GH sample. IGF-I levels were assayed using a radioimmunoassay kit (Immunotech 3516). The detection limit was 3 µg/l and the intra- and interassay coefficients of variation were > 7·4% and 15·5%, respectively. The normal range was 219–644 µg/l for patients aged 18–30 years, 183–405 µg/l for those aged 31–40 years, 54–336 µg/l for patients aged 41–50 years, 71–284 µg/l for those aged 51–65 years and 45–259 µg/l for patients aged 66–85 years.

In addition to the haematology and biochemical analyses, the safety assessment was based on physical examination at each visit, and gallbladder ultrasonography performed at baseline, and at weeks 28 and 48. Acromegaly symptoms were evaluated at each visit and graded as absent (score = 0), mild (score = 1), moderate (score = 2) or severe (score = 3).

The primary efficacy end-point was the proportion of patients with normal age-adjusted IGF-I levels at study end (week 48 of treatment), defined as a level at or below the upper limit of the normal age-adjusted range.

Secondary objectives included documenting the variation in IGF-I levels from baseline (expressed as a percentage of the upper limit of the age-adjusted normal range); mean IGF-I and GH levels; number of patients with serum GH levels of ≤ 2·5 µg/l or < 1 µg/l; and number of patients with no or reduced clinical signs of acromegaly.

### Data analysis

The primary population for the assessment of efficacy was the intention-to-treat population, which comprised all patients who had received at least one injection of lanreotide Autogel and had data for at least one key efficacy variable (i.e. data for GH or IGF-I levels at any visit after week 0). The safety population included all patients who received at least one injection of lanreotide Autogel. As this was an open-label study, descriptive statistics were used. Efficacy was also assessed for subgroups of patients defined by their baseline characteristics.

## Results

### Patient disposition and baseline disease characteristics

Sixty-three patients were recruited into the study: all patients completed the fixed-dose phase, 59 completed the first titrated-dose phase and 57 completed the entire 48-week study. Six patients withdrew prematurely: three because of adverse events, one due to lack of efficacy and two for other reasons (missing injections during weeks 10–12 for one patient, and time interval was too long between injections for the second patient).

Patients’ baseline characteristics are shown in Table 1. At the time of inclusion, 46 patients (73%) had a macro-adenoma. With regard to previous treatment for acromegaly, 37 patients (59%) had undergone pituitary surgery, 12 (19%) had received pituitary radiotherapy and 49 (78%) had been medically treated.

**Table 1:** Baseline demographic and disease characteristics for the intention-to-treat population

Characteristics	*n* = 63
Mean (SD) age, years	55·3 (12·0)
Mean (SD) weight, kg	84·5 (18·8)
Mean (SD) time since diagnosis, years	7·8 (7·1)
Sex	
*n* (%) male patients	38 (60)
*n* (%) female patients	25 (40)
Previous treatment*	
*n* (%) patients who have had pituitary surgery	37 (59)
*n* (%) patients who have had pituitary radiotherapy	12 (19)
*n* (%) patients who have had pituitary medical treatment	49 (78)
Size of adenoma at diagnosis	
*n* (%) patients with microadenoma	11 (17)
*n* (%) patients with macro-adenoma	46 (73)
*n* (%) patients with adenoma of unknown size	6 (10)

* Percentages add up to more than 100 because patients may have had more than one previous treatment.

### Treatment

Following assessment of GH and IGF-I levels at week 12 (i.e. after three injections of 90 mg), the dose of lanreotide Autogel given at subsequent visits during the first titrated-dose phase was decreased to 60 mg in 11 patients, maintained at 90 mg in 4 patients and increased to 120 mg in 44 patients. By the end of the study, lanreotide Autogel was administered at the 60-mg dose in 9 patients (14%), at the 90-mg dose in 4 patients (6%) and at the 120-mg dose in 46 patients (73%). The remaining four patients withdrew during the fixed-dose phase.

### Efficacy

#### Primary efficacy end-point

Treatment with lanreotide Autogel resulted in normalized IGF-I levels (age-adjusted) in 27 patients (43%, 95% CI 31–55) by the end of the study (Table 2).

**Table 2:** Patients with control of IGF-I levels or serum GH or both, for the intention-to-treat population

	Baseline (*n* = 63)	Week 12 (*n* = 61)		End of study (*n* = 63)					
	Age-adjusted IGF-I								
	Abnormal*	Normal	Abnormal	Normal			Abnormal		
Patients, *n* (%)†	63 (100)	16 (26)	45 (74)	27 (43)			36 (57)		
Dose of lanreotide Autogel (mg)	–	90	90	60	90	120	60	90	120
Patients with both IGF-I and GH values, *n*‡	63	16	45	9	5	12	0	3	34
GH level (µg/l)									
< 1	7	11	8	8	3	6	0	1	10
1–2·5	15	3	25	1	1	5	0	1	18
>2·5	41	2	12	0	1	1	0	1	6

* No results were normal at baseline.

† Last value available.

‡ Last value available from last visit at which both IGF-I and GH values were obtained.

#### Secondary end-points

**Fig. 2:**
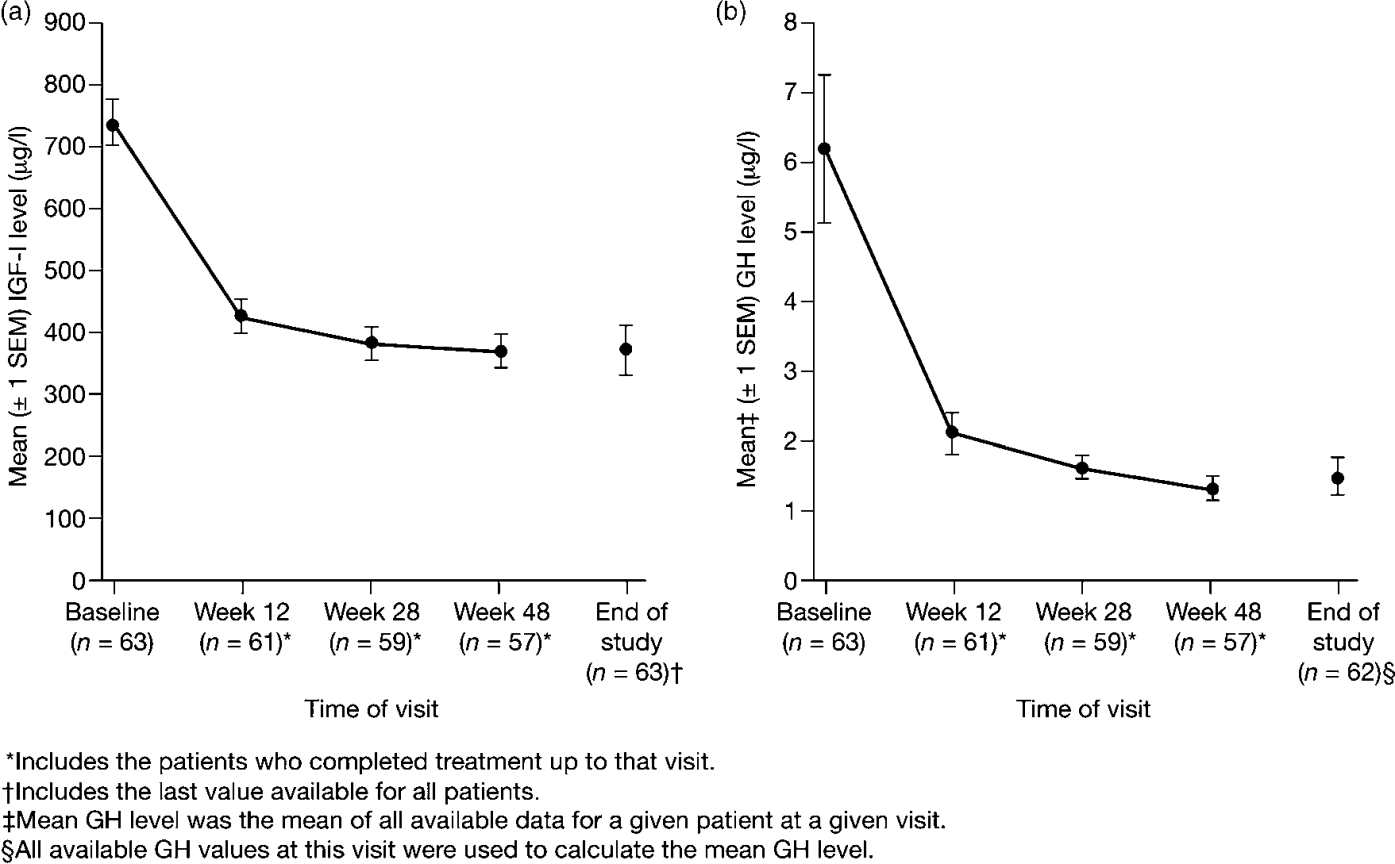
Reduction in mean (SE) levels of serum (a) IGF-I and (b) GH.

Serum IGF-I levels decreased throughout the study period (Fig. 2a) and across all dose groups: during the study mean (SE) IGF-I levels decreased from 432·3 (27·9) µg/l at week 12 to 376·9 (39·9) µg/l at the end of the study. After treatment completion, mean IGF-I levels were 1·3 times the upper limit of the age-adjusted normal range (range 0·3–4·0), compared with 2·5 times the upper limit of the normal range at baseline (range 1·3–5·8).

When normalized IGF-I levels were analysed according to baseline characteristics, there was no influence of gender or prior radiotherapy on the proportion of patients responding.

Normalization of IGF-I levels was accompanied by a marked decrease in mean (SE) GH levels, from 6·2 (1·1) µg/l at baseline to 1·5 (0·3) µg/l at study end (Fig. 2b). At the end of the study, more patients in the 60- and 90-mg dose groups with normalized IGF-I levels had GH levels < 1 µg/l, compared with those who had abnormal IGF-I levels (Table 2). After treatment, GH levels were ≤ 2·5 µg/l in 53 of 62 patients (85%, 95% CI 77–94) and < 1 µg/l in 28 of 62 patients (45%, 95% CI 33–58). One patient did not have postbaseline GH data.

Twenty-four patients (38%) had both normal IGF-I levels and GH levels of ≤ 2·5 µg/l, and 17 of 63 (27%) had both normal IGF-I levels and a GH level of < 1 µg/l. Only two patients had GH levels > 2·5 µg/l. Of the patients who maintained supranormal IGF-I levels, 30 of 37 (81%) achieved GH levels ≤ 2·5 µg/l.

**Fig. 3:**
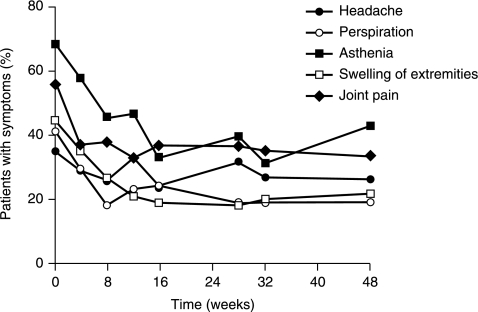
Proportion of patients with acromegaly symptoms by visit over duration of study.

Reductions in IGF-I and GH levels were associated with improvements in acromegaly symptoms throughout the study (Fig. 3). Asthenia was the most frequent acromegaly symptom at baseline. By the end of the study, it had improved in 27 of 43 (63%) patients, and was absent in 36 of 63 (57%) patients, compared with 19 of 62 (31%) at baseline. Similarly, joint pain improved in 27 of 35 (77%) patients who recorded this symptom at baseline. Swelling of extremities, excessive perspiration and headache also improved at the end of the study in 23 of 29 (79%), 21 of 26 (81%) and 15 of 22 (68%) patients, respectively.

### Safety

The most common adverse events were gastrointestinal in nature, with 36 (57%) and 17 (27%) patients reporting at least one episode of diarrhoea or abdominal pain, respectively, over the 48-week study. Liver and biliary system disorders occurred in 16 patients (25%), with cholelithiasis reported in 10 (16%) and other gallbladder disorders in 6 (10%). Ultrasonography, which was performed on 47 patients at baseline and at the end of the study, revealed that there were eight new cases (17%) of gallstones and four new cases (9%) of gallbladder sludge at the end of the study. No cholecystitis or cholangitis were observed.

Twenty-one patients (33%) at baseline and 25 (40%) at the end of the study had fasting blood glucose values > 6·5 mmol/l. Adverse events related to glycoregulation were reported in four patients: in one patient diabetes control worsened, one developed diabetes mellitus and two had hypoglycaemia.

The majority of adverse events were mild (111 events in 18 patients, 29%) or moderate (78 events in 25 patients, 40%) in severity. Thirteen patients experienced serious adverse events during the study, but only one was considered possibly related to the study drug. This case of severe thrombophlebitis led to the early withdrawal of the patient. Two other patients withdrew from the study because of adverse events possibly or probably related to the study drug (injection site pain in one and diarrhoea with abdominal pain in the other).

## Discussion

In this population of 63 patients with acromegaly who had been treated with lanreotide Autogel, normalization of IGF-I levels was demonstrated in 43% of patients who all had abnormal IGF-I levels at study entry. This is comparable with a previous study in which 50% of patients given titrated doses of lanreotide Autogel achieved IGF-I normalization.^9^ Although other studies cite slightly higher percentages of patients achieving normalization in IGF-I levels,^15^ it should be emphasized that, in contrast to other studies, this was the primary end-point in our study and the data analysis was based on the intention-to-treat population, recognized as the most realistic unselected method of assessing the data. Additionally, strict entry criteria were used to ensure that patients had active acromegaly – that is, IGF-I levels increased at least 1·3 times the upper limit of the age-adjusted normal range. In contrast, in the other studies patients were selected on the basis of either GH level or marginally increased levels of IGF-I only.^15^ This may be of importance when interpreting the results, as the response is generally better when baseline GH levels are not highly elevated.^15^ Furthermore, although the inclusion criteria for this study were based on IGF-I levels only, all except one patient met the Cortina consensus criteria for active acromegaly (random GH > 0·4 µg/l and IGF-I levels above the age- and sex-adjusted normal range).^16^ The proportion of patients achieving normalized IGF-I levels in this study was not influenced by gender or prior radiotherapy.

In this study, GH levels ≤ 2·5 µg/l were achieved in 85% of patients, higher than the percentage reported (68%) for titrated-dose lanreotide Autogel.^9^ GH levels < 1 µg/l were achieved in 45% of patients, a higher proportion than achieved with octreotide treatment in a previous study (27%).^17^

This study was conducted in accordance with recent guidelines, which recommend assessing GH and IGF-I control using clearly defined ranges that were updated following recent advances in biochemical assay technologies.^1^ The criteria for biochemical control of acromegaly that will best reduce disease-related morbidity are debated, and there remains no consensus regarding the criteria that predict a response to treatment. GH levels continue to have relevance as a disease marker, and may be the best predictors of the subtle GH excess or early GH dysregulation that is a precursor to acromegaly recurrence in some patients.^18^ Several studies have shown that achieving normal IGF-I levels is a good prognostic factor for acromegaly in terms of morbidity,^19^–^21^ but also for mortality.^3^,^22^ Other studies, however, do not support the link between IGF-I level and excess mortality.^23^,^24^ One recent study supports the validity of the currently used biochemical criteria for assessing long-term mortality and morbidity, but suggests that they may be insufficient in addressing patient quality of life.^25^ Therefore, the association between the improved hormonal control, in terms of both IGF-I and GH level, and the decrease in incidence of acromegaly symptoms in this study reinforces the concept of IGF-I as a useful disease marker.

Discordant results in the achievement of both GH levels ≤ 2·5 µg/l and normal IGF-I levels are frequently observed when treating acromegaly patients with somatostatin analogues,^17^,^26^ and also after surgery.^27^ Similarly, in our study there were 30 patients with GH levels ≤ 2·5 µg/l but high IGF-I levels, and two patients with normal IGF-I levels but high GH levels. Reasons for why such discordance might occur in some patients have been proposed.^17^ There is general agreement that achieving GH levels ≤ 2·5 µg/l is not sufficient for the disease to be considered controlled;^28^ it is thought that the persistence of a continuous, albeit low, hypersecretion of GH (which can be detected with very sensitive assays) may promote the increased IGF-I levels seen in some patients.^28^ The situation whereby patients have normal IGF-I levels but high GH levels occurs less frequently.^29^ It may be related to the fact that IGF-I levels are not only dependent on GH levels, but also on other factors which may interfere with sensitivity to GH at the receptor or the postreceptor level (e.g. the levels of oestrogens, the general nutritional state or the presence of diabetes).^29^,^30^ A direct peripheral effect of somatostatin analogues at a hepatic level has also been suggested to control the GH–IGF-I axis, and may provide an explanation for the discordant GH and IGF-I suppression found in some patients receiving these drugs.^31^

In this study, following a starting dose of 90 mg of lanreotide Autogel, doses were titrated up to 120 mg or down to 60 mg according to GH and IGF-I levels, and therefore hormonal responsiveness. In our experience, although the initial dose of lanreotide Autogel does not appear to affect the rates of hormonal response achieved at optimal control after dose titration, initiation of treatment at 90 mg may result in lower annual cumulative GH concentrations. Thus, the 90-mg dose represents the optimal starting dose. Notably, Ashwell *et al*.^10^ also used 90 mg as a starting dose – patients were switched from long-acting octreotide to 90-mg lanreotide Autogel, with the finding that acromegaly was at least as well controlled by lanreotide Autogel as it had been previously.

Fewer patients receiving the 60-mg dose had abnormal GH and/or IGF-I levels compared with those receiving higher doses, indicating that the patients whose doses are titrated down are the best responders and therefore able to achieve good control with a low dose. This improved efficacy at lower doses has been demonstrated,^8^ and suggests that patients vary in their sensitivity to lanreotide Autogel. Like somatostatin, somatostatin analogues bind to receptors (sst) present on the cell surface of target organs. Five subtypes (sst1–5) have been described, and the currently available somatostatin analogues bind with high affinity to sst2 and sst5. Partial or total resistance of GH-secreting adenomas to somatostatin analogues is in part due to variable tumoural expression or reduced receptor density of the five known sst subtypes.^32^

The majority of patients had their doses titrated up, but 14% responded sufficiently to be able to have their dose titrated down to the lowest dose. The dose-titration frequency (12-week intervals) employed in this study is used widely in clinical practice – generally the dose of lanreotide Autogel is increased when the GH level is > 2·5 µg/l or the IGF-I level is outside of the age-adjusted normal range. The results are consistent with year-long dose-titration studies of other somatostatin analogues.^9^,^30^ An alternative to changing the dose is to change the frequency of dosing;^33^,^34^ for example, it has recently been reported that patients whose acromegaly is well-controlled with lanreotide Autogel 120 mg every 4 weeks may be able to have their dose interval extended to every 6–8 weeks without loss of efficacy.^35^,^36^

The general safety of repeated injections of lanreotide Autogel was assessed using standard haematology and biochemistry tests, physical examination and adverse event reporting. Treatment was well tolerated in this 48-week study and, as expected with somatostatin analogues, the adverse effects were predominantly minor, involving transient gastrointestinal problems, such as diarrhoea and abdominal pain. There is an increased tendency for gallstone formation during long-term somatostatin analogue treatment, but gallbladder echocardiography performed at the end of this study revealed only a slight increase in the presence of gallstones or sludge (eight and four new cases, respectively). There were no cases of complicated gallstones. As a result of their inhibition of the secretion of insulin and glucagon, treatment with somatostatin analogues may be associated with disturbance of glucose control. In the present study, the proportion of patients with clinically significant abnormal glucose values was slightly higher at the end of the study (40%) compared with at baseline (33%), although adverse events of glycoregulation were mixed (two cases of new or aggravated diabetes mellitus and two cases of hypoglycaemia). It is recommended that blood glucose levels are monitored when lanreotide treatment is started, or when the dose is altered and any antidiabetic treatment adjusted accordingly.

## Conclusion

By the end of this 48-week study of lanreotide Autogel, in which IGF-I was used as the primary efficacy criterion, 43% of patients with acromegaly had normalized age-adjusted levels. This was accompanied by control of GH to levels ≤ 2·5 µg/l in 85% of patients and reduction in acromegaly symptoms. Lanreotide Autogel was well tolerated with the safety profile expected of a somatostatin analogue.
